# Retraction: Cell-Substrate Interactions Feedback to Direct Cell Migration along or against Morphological Polarization

**DOI:** 10.1371/journal.pone.0200986

**Published:** 2018-07-19

**Authors:** The PLOS ONE Editors

Following publication of this article [1], the journal was notified of concerns about Figures 2, 3, 4, and 5:

- Figs 3A and 4A were published previously as Figure 3 in [2]. The *Analytical Chemistry* article was not cited in the *PLOS ONE* article; these figures were included in the *PLOS ONE* article without attribution to or permission of the original publication.
- In Fig 5, the ‘tear’ stamp pattern shown vertically on the left side of the image for CA-Cdc42, Pattern B, 18 hours, is vertically inverted and in a different position as compared to the placement of the tear stamp detectable on the original micrograph.
- Concerns were raised about whether Golgi staining in Fig 2 accurately represents the primary data.

The University of Cincinnati has completed an investigation of this work and recommended retraction based on the above concerns.

In line with the University of Cincinnati’s recommendation, the *PLOS ONE* Editors retract this article, as the concerns about these figures call into question the validity of the results and conclusions reported in the article.

GK, C-CH, and CCC agree with the retraction.

## References

[1] Kumar G, Ho C-C, Co CC (2015) Cell-Substrate Interactions Feedback to Direct Cell Migration along or against Morphological Polarization. PLoS ONE 10(7): e0133117 10.1371/journal.pone.0133117 26186588 PMC4506050

[2] Co Carlos C, Ho Chia-Chi, and Kumar Girish (2012). Motility-Based Cell Sorting by Planar Cell Chromatography. Analytical Chemistry 84(23), 10160–10164. 10.1021/ac302855m 23140541 PMC3517722

